# The Effect of Sample Preparation Techniques on Lignin Fourier Transform Infrared Spectroscopy

**DOI:** 10.3390/polym15132901

**Published:** 2023-06-30

**Authors:** Fredrik Heen Blindheim, Jost Ruwoldt

**Affiliations:** RISE PFI AS, Høgskoleringen 6B, 7491 Trondheim, Norway; fredrik.heen.blindheim@rise-pfi.no

**Keywords:** FTIR, attenuated total reflectance, KBr pellet, lignin, kraft lignin, soda lignin, non-aqueous potentiometric titration, size exclusion chromatography, lignin characterization

## Abstract

The characterization and quantification of functional groups in technical lignins are among the chief obstacles of the utilization of this highly abundant biopolymer. Although several techniques were developed for this purpose, there is still a need for quick, cost-efficient, and reliable quantification methods for lignin. In this paper, three sampling techniques for fourier transform infrared (FTIR) spectroscopy were assessed both qualitatively and quantitatively, delineating how these affected the resultant spectra. The attenuated total reflectance (ATR) of neat powders and DMSO-*d6* solutions, as well as transmission FTIR using the KBr pelleting method (0.5 wt%), were investigated and compared for eight lignin samples. The ATR of neat lignins provided a quick and easy method, but the signal-to-noise ratios in the afforded spectra were limited. The ATR of the DMSO-d6 solutions was highly concentration dependent, but at a 30 wt%, acceptable signal-to-noise ratios were obtained, allowing for the lignins to be studied in the dissolved state. The KBr pelleting method gave a significant improvement in the smoothness and resolution of the resultant spectra compared to the ATR techniques. Subsequently, the content of phenolic OH groups was calculated from each FTIR mode, and the best correlation was seen between the transmission mode using KBr pellets and the ATR of the neat samples (R^2^ = 0.9995). Using the titration measurements, the total OH and the phenolic OH group content of the lignin samples were determined as well. These results were then compared to the FTIR results, which revealed an under-estimation of the phenolic OH groups from the non-aqueous potentiometric titration, which was likely due to the differences in the p*K*_a_ between the lignin and the calibration standard 4-hydroxybenzoic acid. Further, a clear correlation was found between the lower $M_{n}$ and the increased phenolic OH group content via SEC analyses. The work outlined in this paper give complementary views on the characterization and quantification of technical lignin samples via FTIR.

## 1. Introduction

Lignin is the world’s second most abundant biopolymer and is the most plentiful source of natural polyphenolics [1]. Today, lignin is mainly obtained as a by-product of paper manufacturing processes and is mostly burned as a low-effect fuel and used for chemical recovery in the mills [2]. There is a severe under-utilization of an already available natural resource, and if better usages are to be industrially viable, insight into both the modification and analysis of lignin is vital.

Lignin is primarily comprised of the following three phenylpropene units (lignols): *p*-coumaryl alcohol (H unit, 4-hydroxyphenyl), coniferyl alcohol (G unit, guaiacyl) and sinapyl alcohol (S unit, syringyl) [3]. It is common for these monolignols to have a 4-hydroxyphenyl unit, where G and S contain one or two methoxy groups *ortho* to the hydroxyl group, respectively [4]. The composition of lignins varies widely with its source; e.g., grass lignin contains a mix of the three monolignols, while gymnosperm (softwood) lignin contains mostly coniferyl (G) units, and angiosperm (hardwood) lignin contains mostly G and S units [5]. The manner of bonding between the monolignols gives rise to a highly irregular structure, containing functional groups such as ethers, carboxylic acids, methoxy groups, and both aliphatic and aromatic hydroxyl groups. Technical lignins are sorted into two groups, where the major group comprises the sulfur-containing lignins from the kraft or sulfite pulping processes. During depolymerization, these are exposed to elevated temperatures and harsh cooking liquors that can lead to crosslinking and larger molecular size lignins compared to other technical lignins. The second group encompasses the non-sulfur-containing technical lignins, such as the soda/anthraquinone and organosolv lignins. The soda pulping process is somewhat oxidative and yields lignins with higher contents of carboxylic acids. The milder organosolv pretreatment generally produces lignins with a smaller molecular size, but of high purity and more concise molecular weight distributions [6,7].

Today, the use of lignin as a raw resource is explored in, e.g., copolymers and emulsifiers [5], as free radical scavengers [8], and as a precursor for the production of various fine chemicals [5,9]. Various methods of lignin activation were also explored for tuning the biopolymers’ reactivity for the development of more specialized chemicals, such as hydroxypropylation with epoxides or cyclic carbonates [10,11], ureathenation with isocyanides, phenolation, and nitration [12]. Particular interest has been placed on lignin as a potential source of high-value chemicals like BTX (benzene, toluene, xylenes) and phenols, or as other high-value products such as carbon fiber, activated carbon, or composite materials [13]. An important step in the utilization of lignins for such higher value applications is the ability to perform correct analyses and characterizations, preferably by using low-cost techniques that are readily available.

The characterization and quantification of lignins have been central pursuits in lignin science for some time, and common procedures include costly ^31^P-NMR [14], destructive techniques like thermogravimetric analyses and pyrolysis [15], or UV–Vis analyses [16]. To quantify the abundance of specific functional groups in lignin samples, specialized techniques are often used; e.g., hydroxyl groups and carboxylic acids can be quantified via ^31^P- and ^13^C NMR [17], and the identification of methoxy groups has been performed using ^13^C NMR [18]. However, due to cost and practicalities, the use of destructive “wet chemical” methods is still common practice [19].

As an alternative to labor-intensive “wet chemical” methods of characterizing lignin samples, FTIR has also long been a popular analysis technique due to its speed and relatively low cost [8,20,21]. It is often used for qualitative analyses [18,22], but in combination with chemometric methods like principal component analysis (PCA) and partial least squares (PLS) regression, FTIR can be used quantitatively. Examples of this include the compositional analysis of lignocellulosic biomass [21], or the monitoring of the conversion of hydrolysis reactions [23]. Transmission FTIR using KBr pellets has largely been replaced with attenuated total reflectance (ATR), partly due to its ease of use and non-dependency on particle size, but also due to less water absorbance caused by the hygroscopic KBr [24,25]. Additionally, ATR is compatible with liquid samples or solutions, which could be advantageous for, e.g., forming more homogeneous mixtures of polymers with possibly increased accessibility to functional groups. Still, some advantages remain with the pelleting technique, e.g., information about the bulk material is obtained instead of only surface effects for inhomogeneous samples [26], and higher reproducibility can be obtained between parallels [27]. In this work, the FTIR analyses conducted using the transmission-approach were compared to the ATR of neat powders and solutions. Through both qualitative comparisons and quantifications of the phenolic OH groups of the lignin samples, these three sampling techniques were assessed and compared with other techniques. The knowledge obtained in this study therefore benefits the improved quantitative analyses for lignin samples via FTIR.

## 2. Materials and Methods

### 2.1. Chemicals and Lignin Samples

All reagents and solvents were purchased from Sigma-Aldrich (MERCK, Oslo, Norway) and were used without further purification. The softwood kraft lignin samples BioPiva 100 and BioPiva 395 were obtained from UMP Biochemicals, Helsinki, Finland, and the Lignoboost sample was provided by Nordic Paper/RISE LignoDemo plant in Bäckhammar, Sweden. Three Arkansas/straw soda lignin samples (Protobind 1000, 2000, and 6000) were purchased from PLT Innovations, Zürich, Switzerland. The soda lignin and organosolv lignin samples were produced from Norwegian spruce as described by Ruwoldt and Tanase Opedal [28].

### 2.2. Acetylation of Lignin Samples

All lignin samples were dried under vacuum at 55 °C for 5 h prior to modification, with periodical flushing of N_2_. Lignin (1 g) was dissolved in a stirring mixture of DMF (10 mL), pyridine (10 mL), and acetic anhydride (10 mL) at ambient temperature. After 48 h, the dissolved and acetylated lignins were poured into water (0 °C, 400 mL) and filtrated using 0.1 µm VVP Millipore filters before the filtrate was washed with distilled water (3 × 300 mL). The retrieved lignin was dried for at least 24 h under ambient conditions before it was further dried at 55 °C under vacuum for 5 h.

The individual analysis types are described in detail below. For better overview, Table 1 lists the different analysis types and the lignin raw materials these analyses were conducted on.

### 2.3. FTIR Sample Preparation

The solvated analytes were prepared by mixing lignin samples (10–50 wt%) with DMSO-*d_6_*, followed by sonication for 15 min. The solutions were applied directly onto the ATR crystal. The KBr pellets were prepared by mixing dried KBr (at 120 °C for at least 1 h) with lignin samples (0.5 wt%), followed by grinding and then pressing the powder into a disk at a pressure of 10 tons for 2 min. Neat powders were applied directly to the ATR crystal and were pressed down with equal force.

### 2.4. FTIR Spectroscopy

The FTIR spectra were obtained using a PerkinElmer Spectrum 3 MIR spectrometer equipped with an MIR TGS (15,000–370) cm^−1^ detector, a Universal KBr disc holder, and a Universal Attenuated Total Accessory (UATR) module. ATR samples were pressed onto a composite of ZnSe and diamond using a torque arm, ensuring equal pressure was applied to all samples. Spectrum recording was performed using the PerkinElmer Spectrum IR v 10.7.2.1630 software. All spectra were recorded as two parallels between 4000 and 500 cm^−1^, with 4 cm^−1^ resolution and data point collection and with the average of 64 scans each. Each sample was analyzed twice, yielding four measurements per point. For analyte solutions and KBr pellets, the background was measured on blank solutions or KBr disks, respectively. The FTIR data were processed via manual baseline correction using a cubic spline function. The baseline-corrected data were further normalized via the aromatic stretching band at 1505–1510 cm^−1^ for better interlaboratory comparison [26,29].

### 2.5. Total OH Content Determination Using the Consumed Acetic Acid Calculation Method

The content of aliphatic OH groups was determined by using an adapted method from the ISO standard (ISO 14900:2017) for measuring the hydroxyl numbers in polyols [30]. Dried lignin samples (1 g) were dissolved in DMF (10 mL), pyridine (10 mL), and acetic anhydride (10 mL) and were stirred at ambient temperature. After 48 h, samples (1 mL) were collected and cooled in an ice bath, while distilled water (5 mL) was added to each sample to hydrolyze the remaining acetic anhydride. After further reacting for 1 h, the ice bath was removed, and the samples were left for 1 h at ambient temperature. Afterwards, 5 mL of 1 N phosphoric acid was added to the sample to precipitate any lignin remaining in the solution, and phosphoric acid acted as a buffer for improved titration. A defined amount of sample was then filtrated through a 0.45 µm PTFE syringe filter, diluted with distilled water (300 mL), and titrated to pH 8 with 0.1 N NaOH. The total OH was calculated from the amount of acetic acid consumed during acetylation, i.e., the difference between the blank titration and the titration of solvent sampled from the acetylated lignin reagent.

### 2.6. Non-Aqueous Potentiometric Titration

The carboxyl and phenolic hydroxyl groups of lignin samples were determined via non-aqueous titration, which followed the procedure by Dence et al. [31] as modified by Gosselink et al. [32]. In short, lignin (150 mg) and 4-hydroxybenzoic acid (20 mg) were first dissolved in DMSO (60 mL) and then titrated with a solution of 0.05 N tetrabutylammonium hydroxide (TBAH) in methanol (100 mL) and 2-propanol (1900 mL). The exact molarity of the titrant was determined via titration of 50 mg benzoic acid (50 mg). During each measurement, the inflection points near −350 mV and −500 mV were correlated with the ionization of carboxyl and phenolic hydroxyl groups of lignin, respectively, while also subtracting the contribution of the internal standard, 4-hydroxybenzoic acid. Each sample was run in triplicate.

### 2.7. SEC Analyses

Size exclusion chromatography (SEC) was conducted on an Agilent 1260 Infinity II system equipped with an auto sampler, column oven, and RI detector. Two Agilent PolarGel-L columns were used in series at a temperature of 50 °C. DMSO with LiBr (1 g/L) as background electrolyte was used as the mobile phase. Analytical samples were prepared by dissolving lignin (5 g/L) in the same solution as the mobile phase. The mobile phase was pumped at 1 mL/min, yielding a back pressure of approximately 140 bar. Calibration was performed using PEG/PEO standards of known molecular weight (250–200,000 Da in increments), which were purchased from Sigma-Aldrich, Norway. The calibration line obtained using these standards was fitted via a fourth-degree polynomial function. Each elution profile was also corrected by subtracting the baseline, i.e., the linear fit of data, before and after sample elution. The column’s higher cut-off was given as 60,000 Da. Peak fronting above 100,000 Da was indeed observed for some of the samples, as the column separation was no longer in the linear logarithmic regime. These data were hence omitted from the evaluation.

The number and mass average molecular weight $M_{n}$ and $M_{w}$ were calculated according to Equations (1) and (2), respectively, where $M_{i}$ denotes the calibrated molecular weight, and $N_{i}$ is the detector signal at instance i.

(1) $$M_{n}=\frac{\sum M_{i}N_{i}}{\sum N_{i}}$$

(2) $$M_{w}=\frac{\sum M_{i}^{2}N_{i}}{\sum M_{i}N_{i}}$$

## 3. Results

### 3.1. Comparison of Sample Preparation Techniques

**Figure 1:**
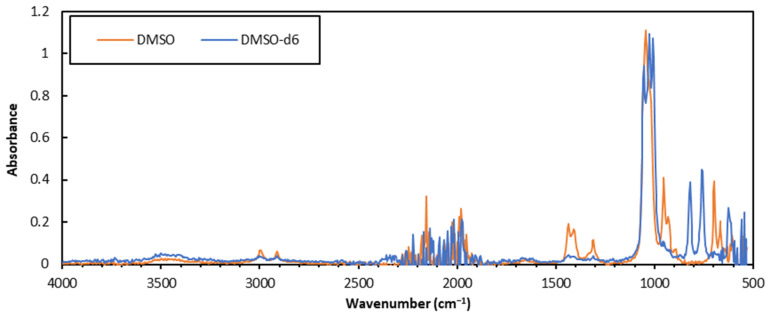
Comparison of the ATR-FTIR spectra of DMSO and DMSO-*d6*.

Although sparse, some work on the solution-state FTIR of lignins was previously reported in solvents such as chloroform or in alkaline aqueous solutions [26]. Both types of analyses gave a high peak resolution but had individual shortcomings either in the solubility profile or in health, safety, and environment (HSE) concerns. Aqueous solutions will not dissolve acetylated or alkylated lignin samples, and interactions between deprotonated functionalities and cations in the solution were observed, which could complicate the comparison of different samples. On the other hand, chloroform performs well with acetylated lignins, but will not dissolve the non-acetylated material [26], and due to the likely carcinogenic properties of the solvent, safer options are preferred. Other solvents that can dissolve acetylated lignins were considered such as DMF, 1,4-dioxane, and THF [33]. However, due to interfering signals, e.g., from the carbonyl C=O stretching of DMF or the crowded C-H stretching band at 2800–3000 cm^−1^ of THF [34], these solvents were not utilized further. Instead, DMSO was seen as an attractive solvent both due to it being relatively safe to handle, but also due to its ability to dissolve both acetylated and non-acetylated lignin samples [33]. The S=O stretching vibration of DMSO appears at 1044 cm^−1^ and will therefore not overlap with any C=O vibrations of interest in the analytes. After a comparison of pure DMSO and the deuterated DMSO-*d6*, a small shift in the S=O stretch from 1044 to 1024 cm^−1^ was seen, but more importantly, lower intensities were observed for the signals in the 1250–1500 cm^−1^ range (see Figure 1). This would lead to less interference of the analyte signal bands in this range. Little difference was observed between the two solvents in the C-H and O-H stretching regions (for lignin, 2840–3000 cm^−1^ and 3412–3460 cm^−1^ [26,34]).

**Figure 2:**
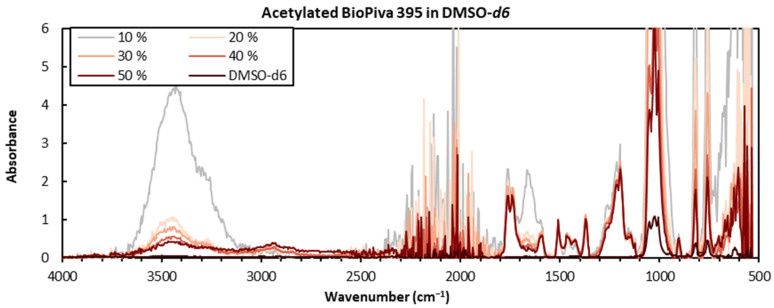
Overlaid spectra of acetylated BioPiva 395 kraft lignin in DMSO-*d6* at different concentrations (in wt%) and blank DMSO-*d6*. The spectra were baseline corrected and normalized to 1508 cm^−1^.

Subsequently, the effect of different concentrations of lignin in DMSO-*d6* on the resulting FTIR spectra was investigated. The results indicate that at lower concentrations, the contaminants would dominate the spectra, making these less suitable for quantitative or semi-quantitative purposes (see Figure 2). This was particularly clear for the 10 wt% sample, where presumably small amounts of absorbed water vapor gave an exaggerated O-H stretching band at ca 3100–3600 cm^−1^ and an H-O-H scissoring band at 1668 cm^−1^. This effect was amplified by the performed normalization to the aromatic C-H signal found at 1500–1510 cm^−1^ [34]. By increasing the analyte concentration to 30 wt%, the signal-to-noise ratio was increased enough to largely suppress this effect. While the sample preparation was acceptable at this concentration, it became challenging and strenuous at 40% and 50% concentrations and resulted in inhomogeneous solutions even after extended sonication treatments. These were deemed unsuited, while the 30 wt% concentration sample gave an acceptable resolution and signal-to-noise ratio.

**Figure 3:**
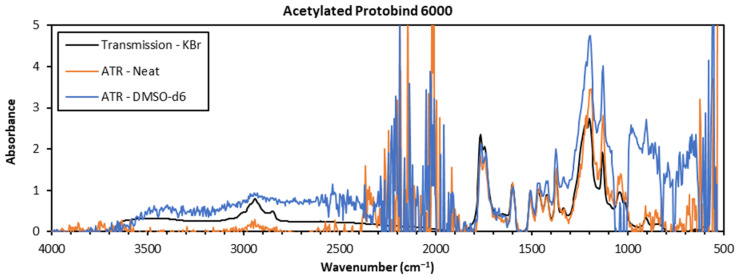

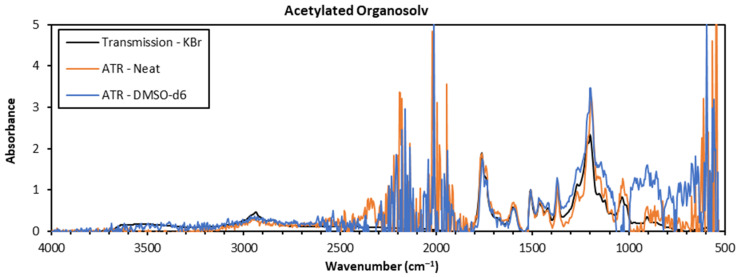
Overlaid spectra of acetylated Protobind 6000 (**top**) and organosolv (**bottom**) lignins recorded via transmission (KBr pellets) and ATR (neat and solution-state) FTIR.

When the transmission KBr pelleting technique was compared to neat or solution-state ATR, several differences were apparent (see Figure 3). First, the absence of the large noise band at 1900–2400 cm^−1^ was noted, which was prominent in the spectra of both ATR techniques. Further, the baselines produced from the KBr pellet transmission spectra were surprisingly smooth in comparison to the ATR spectra across the whole spectrum. In contrast, the ATR spectra do produce smooth spectra, but only in the 1100–1800 cm^−1^ range, although this could still be useful for comparisons towards spectral reference libraries. However, at wavenumbers above 2800 cm^−1^, the baseline was so jagged that information on the C-H and O-H stretching bands could be misrepresented. A lower intensity at higher wavenumbers is one of the innate effects associated with ATR due to the decreased penetration depth of the evanescent wave [35], which, in this case, resulted in non-viable spectra. Albeit a better sensitivity at high wavenumbers and a better signal-to-noise ratio were obtained, the transmission FTIR spectra were qualitatively similar to the ATR spectra in the fingerprinting region.

### 3.2. Phenolic OH Group Content of Lignin Samples in Dependence of FTIR Measuring Mode

To assess and compare the transmission and ATR-FTIR modes in an applicative setting, the phenolic OH group contents of eight lignin samples were calculated. The lignin samples were first acetylated with acetic anhydride and pyridine as described in Section 2.2. The FTIR analyses of the acetylated lignins then allowed for the phenolic OAc and aliphatic OAc groups to be distinguished by their respective ester C=O stretching vibrations at 1764 and 1744 cm^−1^. By using Equation (3), published by Wegener and Strobel [36], the content of the phenolic OH groups can be expressed in mmol per gram lignin as follows:

(3) $$C_{Phenol-OH}=5.60\frac{IR_{1765}}{IR_{1745}}-1.21$$

**Figure 4:**
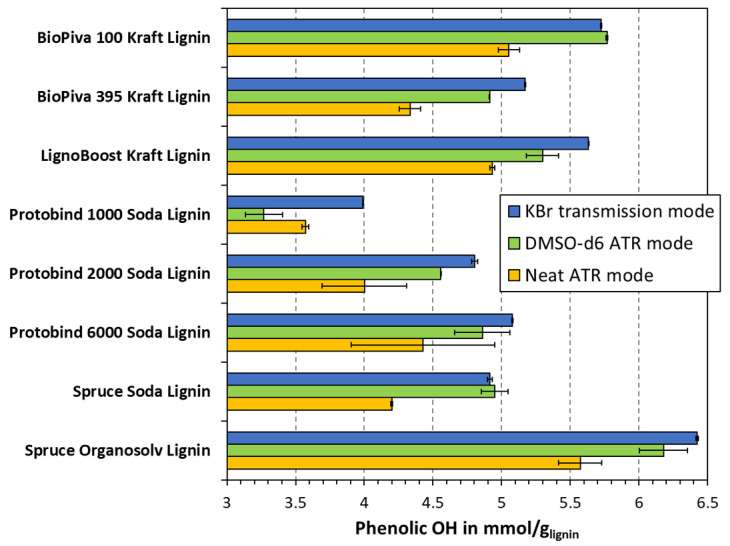
The effect of measuring mode on calculated phenolic OH group content from FTIR spectroscopy.

The average values for the eight lignin samples are plotted in Figure 4. The three different sample preparation techniques gave some variety in the calculated phenolic OH group contents, and different levels of reproducibility were seen between the parallels of the methods. An overall greater precision was obtained between the KBr pellet transmission samples than for the ATR techniques, as indicated by a lower standard deviation. In absolute terms, the arithmetic means of the standard deviation for the eight measurements are 0.012, 0.225, and 0.269 for KBr, DMSO-d6, and neat, respectively. The standard deviation of the transmission mode measurements was hence 20 times lower than that for the ATR. This highlights the better reproducibility obtained using the transmission mode KBr method.

**Figure 5:**
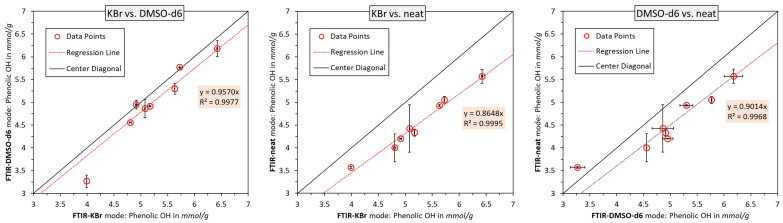
Correlation of calculated phenolic OH group contents using three measuring modes. DMSO-d6 vs. KBr (**left**); neat vs. KBr pellets (**middle**); neat vs. DMSO-d6 (**right**). Error bars mark the min/max values.

To enable the comparison between the three measuring modes, the results are further graphed and compared in Figure 5. In the case of perfect agreement between the two modes, all of the data points would lie on the center diagonal. The correlation ATR-FTIR in the DMSO-*d6* and transmission mode (KBr) were the best, as the measured values were closest to the center diagonal. In addition, the regression line is the closest to the center diagonal, with a slight skewing towards the higher values detected in the KBr. The neat lignin analyzed via ATR-FTIR gave consistently lower values than the other approaches. This deviation was, on average, higher at an elevated phenolic OH content, indicating a proportional skewing towards one side. In other words, the ATR-FTIR of neat lignin yielded an underestimation of the phenolic OH content, as compared to the other two approaches. Judging by the correlation between the KBr and DMSO-*d6* modes, it could be concluded that the ATR-FTIR of neat lignin was the least accurate method. This would further be supported by theory as the latter method measures predominantly surface information, whereas the other two modes probe the whole lignin macromolecule. The preparation of lignin particles is conducted by precipitation from the solution, which could yield a stearic orientation within the colloidal structure, i.e., an inhomogeneous or non-representative distribution of functional groups on the surface. Still, to validate this theory, a comparison with other methods must be conducted. All in all, it appears that the three measuring modes exhibit a close fit with the linear regression model. This suggests that one measuring mode can indeed be superimposed onto another, using only the correction factor c from the regression line y=c·x, as indicated in Figure 5. Based on the closeness to this regression line, the best correlation was obtained for the KBr method and neat FTIR-ATR, which carried an R value of 0.9995. Still, precision and accuracy must be distinguished; however, it is interesting to note that the correction factor *c* appears to be applicable in all three cases of Figure 5.

### 3.3. Comparison with Non-Aqueous Potentiometric Titration and Total OH

**Figure 6:**
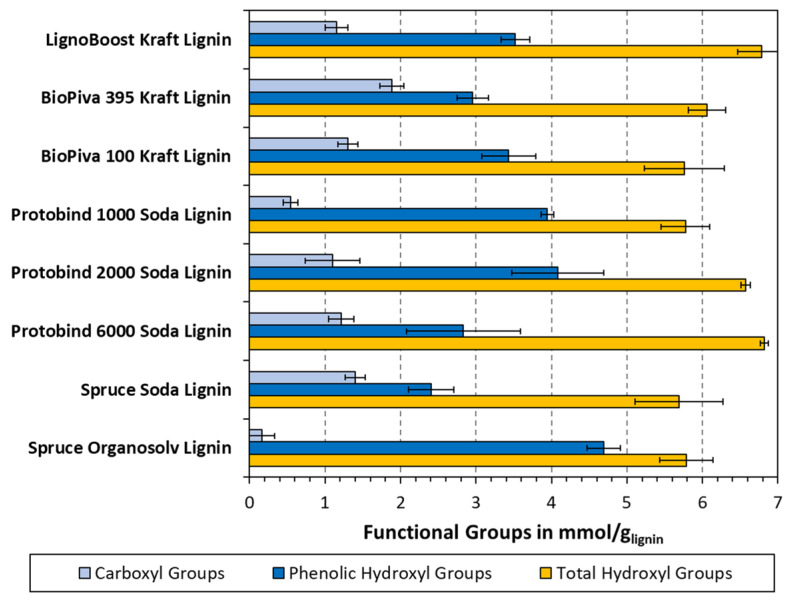
Carboxyl and phenolic hydroxyl groups determined using non-aqueous potentiometric titration. Total hydroxyl groups determined using the measurement of acetic acid consumed during acetylation.

The results from non-aqueous potentiometric titration are plotted in Figure 6, which also comprise the total OH measurements performed using the consumed acetic acid calculation method. The titration measured the contents of both the phenolic OH and carboxyl groups, both of which are important to consider. While the phenolic OH from titration may be directly compared to the FTIR results, the C=O bonds found in the carboxyl groups can skew the FTIR measurements, as these will also contribute to the signal measured at 1764–1744 cm^−1^. The total OH, on the other hand, can be expected to yield higher values than the phenolic OH, as it also comprises portions of aliphatic OH.

A general trend can be seen in Figure 6, where the phenolic OH content is the highest in the organosolv lignin, followed by Protobind 2000 and 1000, then the kraft lignins, and the Protobind 6000 and spruce soda lignin exhibit the lowest content. The trend is less clear for the total OH content, where all the samples ranged between 5.8 and 6.8 mmol/g. The obtained values are in the same general ranges as found using other techniques such as ^31^P or ^13^C NMR, but a direct comparison can be difficult. Balakshin et al. saw fewer differences between the soda, kraft, and organosolv lignins, but in contrast, the “native” milled wood lignins had generally high aliphatic OH and low phenolic OH contents [17].

**Figure 7:**
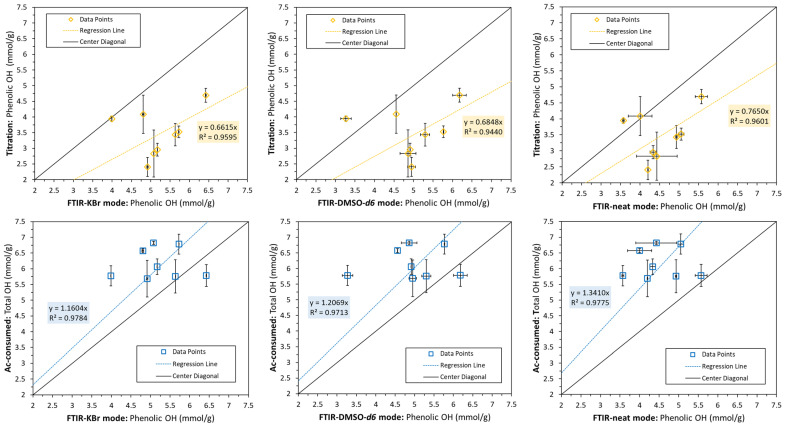
Correlation between phenolic OH (**top**) and total OH groups (**bottom**) determined via non-aqueous titration or the consumed acetic acid calculation method, respectively, with phenolic OH measured using FTIR via three measuring modes. Error bars mark the min/max values.

The results were further correlated with the FTIR measurements shown in Figure 7. Apart from two outliers, the total OH was estimated to be higher than the phenolic OH content. This makes sense, as the total OH must also comprise portions of aliphatic OH. This difference was the greatest for comparing the total OH with the phenolic OH from the FTIR neat mode, and the lowest for the FTIR transmission mode using KBr. The phenolic OH determined via non-aqueous titration was, on the other hand, lower than most measurements performed using FTIR. Similar observations were indeed made by other authors [32]. It is well known that introducing electron-donating substituents in the para and ortho positions of phenols will increase the observed hydroxyl p*K*_a_ value. For instance, phenol has a p*K*_a_ value of 9.98, while *ortho*-methylphenol, *para*-methoxyphenol, and *para*-aminophenol have p*K*_a_ values of 10.14, 10.21, and 10.30, respectively. Additionally, electron-withdrawing groups will reduce the observed p*K*_a_ values; e.g., *para*-nitro or *para*-chlorophenol have p*K*_a_ values of 7.15 and 9.38, respectively [37,38]. There should therefore be a small discrepancy between the p*K*_a_ values of the titration calibration standard, 4-hydroxybenzoic acid, and the lignins’ different hydroxyl groups. This could, in part, explain the deviances between the phenolic OH content obtained via titration and the FTIR methods.

### 3.4. Lignin Molecular Weight Distribution

**Figure 8:**
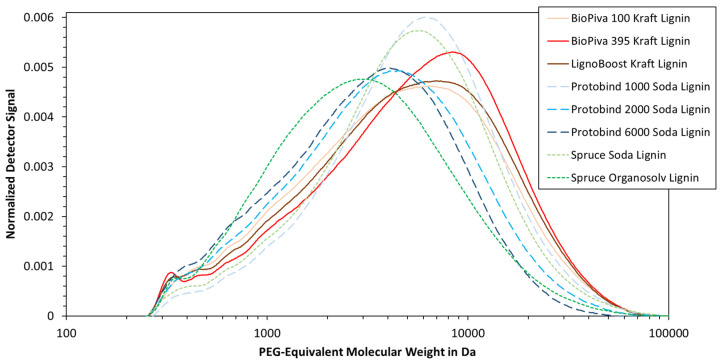
Molecular weight distribution of different lignin samples measured using size exclusion chromatography (SEC).

All eight lignin samples were also analyzed using size exclusion chromatography (SEC), as this can yield complementary information about the molecular weight distribution. As can be seen in Figure 8, most of the samples exhibited their main peak at 2000–9000 Da. This peak was located at the lowest molecular weight for the organosolv lignin, followed by the soda lignin, and the kraft lignin at last. Such trend is indeed in agreement with the data published by other authors [39,40,41].

The molecular weight distribution seen in Figure 8 was converted to the number average ($M_{n}$) and mass average molecular weight ($M_{w}$). The final values are listed in Table 2, which show a similar trend as described above. In particular, the organosolv lignin exhibits the lowest $M_{n}$ values, followed by the Protobind 6000 and 2000 soda lignins. The molecular weight of the spruce soda lignin is on the same level as the kraft lignin samples. This may be due to the larger contribution at a high molecular weight range, as the mass average is based on the second momentum. The polydispersity index (PI) ranges from 2.0 to 2.3, which is low compared to the values in the literature [17,35]. One explanation could be the exclusion of a high molecular weight lignin, as the SEC column has a nominal exclusion limit of a maximum of 60 kDa. Deviations from the literature may also arise from the use of an external standard, as the PEG/PEO standard is a linear polymer, which may not correctly represent the branched lignin macromolecule. The data in Table 2 should hence be interpreted with care.

**Figure 9:**
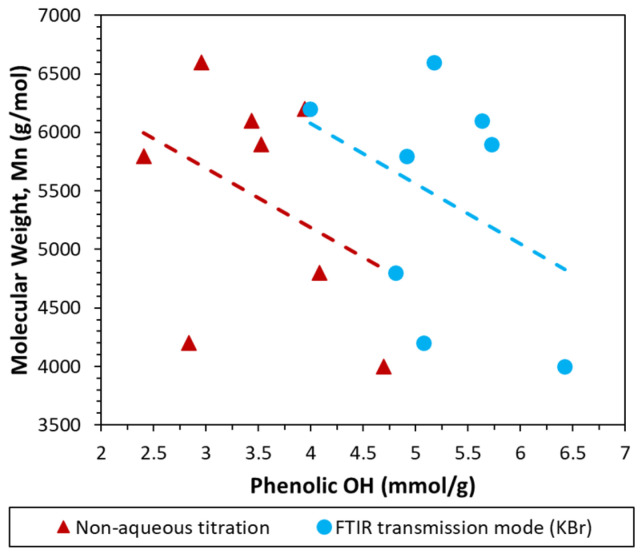
Number average molecular weight (Mn) with respect to phenolic OH measured using two methods.

The depolymerization of lignin also entails that a greater number of functional groups are exposed. A lower molecular weight can hence be encompassed by a greater abundance of phenolic OH, as was shown by some authors [36]. While there are other factors affecting the phenolic OH content as well, the molecular weight may hence be taken as a coarse indicator. Considering lignin samples with similar origin, BioPiva 100 exhibited the lowest molecular weight of all softwood kraft lignin samples, whereas Protobind 6000 showed the same for all Arkansas/straw lignin samples. In each case, these were also the samples with the highest phenolic OH content of their respective type, as measured using FTIR. The number average molecular weight was further plotted against the phenolic OH content in Figure 9. It can be seen that the general trend also indicates a higher phenolic OH at a lower $M_{n}$, which corroborates the findings presented in this article.

## 4. Conclusions

In this study, three FTIR sampling techniques were compared both qualitatively and quantitatively on eight technical lignin samples. The analysis of neat powdered material using ATR was a quick and easy method, but one that gave low signal-to-noise ratios. The resultant spectra also contained a noise band at 1900–2400 cm^−1^, which might interfere with the assessment of the spectra in certain cases. Solution-state ATR was also investigated on kraft lignin samples dissolved in DMSO-*d6*, where lower solvent signal intensities were seen in the 1250–1500 cm^−1^ range compared to the analyses in non-deuterated DMSO. A good signal-to-noise ratio was seen for concentrations of 30 wt%, but higher concentrations gave inhomogeneous solutions. The smoothest spectra with the best signal-to-noise ratios were obtained in transmission mode using KBr pellet samples, which produced a non-jagged baseline with no visible noise band at 1900–2400 cm^−1^.

The quantification of the phenolic OH group content was performed on eight technical lignins by using the three FTIR sampling techniques and non-aqueous potentiometric titration. When comparing the FTIR methods, the highest correlation was seen between the neat ATR samples and the KBr pellet samples, with an obtained R^2^ of 0.9995. Lower results were obtained from the non-aqueous titration compared to the FTIR. A general trend was found for all three FTIR methods, where the organosolv lignin had the highest OH group content, followed by the kraft lignins, and lastly, the soda lignins. From the potentiometric titration, the organosolv lignin still had the highest phenolic OH content, but a less clear grouping was seen between the kraft and soda lignins.

From the SEC analyses, most samples exhibited their main peak at 2000–9000 Da, where the kraft lignins had the highest $M_{n}$ values, and the organosolv lignin had the lowest. Somewhat low polydispersity indices of between 2.0 and 2.3 were obtained, which are attributed to a combination of the exclusion limit of the SEC column and the choice of the linear PEG/PEO calibration standard. Finally, a clear connection was seen between the molecular weight and OH groups of the lignin samples, where lower $M_{n}$ values correlated to a higher phenolic OH content. These findings provide valuable insight into the characterization and quantification of different types of technical lignins and can possibly be useful for the modification of lignins for renewable and green applications.

## Figures and Tables

**Table 1:** Analysis type and type of lignin raw material.

Analysis Type	Lignin Raw Material
Neat FTIR-ATR	Acetylated lignin
FTIR-ATR on lignin in DMSO-d6	Acetylated lignin
Transmission FTIR in KBr	Acetylated lignin
Non-aqueous potentiometric titration	Unmodified lignin
Amount of acetic acid consumed (total OH)	Reaction solvent after acetic anhydride hydrolysis, buffering, and removal of lignin via filtration
Size exclusion chromatography (SEC)	Unmodified lignin

**Table 2:** The calculated number and weight averages of Mn and Mw, and polydispersity index (PI) of the lignin samples.

Lignin	Mn (g/mol)	Mw (g/mol)	PI
BioPiva 100 Kraft Lignin	5900	13,400	2.3
BioPiva 395 Kraft Lignin	6600	13,800	2.1
LignoBoost Kraft Lignin	6100	13,500	2.2
Protobind 1000 Soda Lignin	6200	12,300	2.0
Protobind 2000 Soda Lignin	4800	10,500	2.2
Protobind 6000 Soda Lignin	4200	8100	2.0
Spruce Soda Lignin	5800	12,000	2.1
Spruce Organosolv Lignin	4000	9200	2.3

## Data Availability

The data presented in this study are available upon request from the corresponding authors.
